# Child Mortality Estimation: Appropriate Time Periods for Child Mortality Estimates from Full Birth Histories

**DOI:** 10.1371/journal.pmed.1001289

**Published:** 2012-08-28

**Authors:** Jon Pedersen, Jing Liu

**Affiliations:** Fafo Institute of Applied International Studies, Oslo, Norway; Umeå Centre for Global Health Research, Umeå University, Sweden

## Abstract

**Background:**

Child mortality estimates from complete birth histories from Demographic and Health Surveys (DHS) surveys and similar surveys are a chief source of data used to track Millennium Development Goal 4, which aims for a reduction of under-five mortality by two-thirds between 1990 and 2015. Based on the expected sample sizes when the DHS program commenced, the estimates are usually based on 5-y time periods. Recent surveys have had larger sample sizes than early surveys, and here we aimed to explore the benefits of using shorter time periods than 5 y for estimation. We also explore the benefit of changing the estimation procedure from being based on years before the survey, i.e., measured with reference to the date of the interview for each woman, to being based on calendar years.

**Methods and Findings:**

Jackknife variance estimation was used to calculate standard errors for 207 DHS surveys in order to explore to what extent the large samples in recent surveys can be used to produce estimates based on 1-, 2-, 3-, 4-, and 5-y periods. We also recalculated the estimates for the surveys into calendar-year-based estimates. We demonstrate that estimation for 1-y periods is indeed possible for many recent surveys.

**Conclusions:**

The reduction in bias achieved using 1-y periods and calendar-year-based estimation is worthwhile in some cases. In particular, it allows tracking of the effects of particular events such as droughts, epidemics, or conflict on child mortality in a way not possible with previous estimation procedures. Recommendations to use estimation for short time periods when possible and to use calendar-year-based estimation were adopted in the United Nations 2011 estimates of child mortality.

## Abstract 2

Jon Pedersen and Jing Liu examine the feasibility and potential advantages of using one-year rather than five-year time periods along with calendar year-based estimation when deriving estimates of child mortality.

## Editors' Summary

**Background:**

In 2000, world leaders set, as Millennium Development Goal 4 (MDG 4), a target of reducing global under-five mortality (the number of children who die before their fifth birthday to a third of its 1990 level (12 million deaths per year) by 2015. (The MDGs are designed to alleviate extreme poverty by 2015.) To track progress towards MDG 4, the under-five mortality rate (also shown as _5_ *q*_0_) needs to be estimated both “precisely” and “accurately.” A “precise” estimate has a small random error (a quality indicated by a statistical measurement called the coefficient of variance), and an “accurate” estimate is one that is close to the true value because it lacks bias (systematic errors). In an ideal world, under-five mortality estimates would be based on official records of births and deaths. However, developing countries, which are where most under-five deaths occur, rarely have such records, and under-five mortality estimation relies on “complete birth histories” provided by women via surveys. These are collected by Demographic and Health Surveys (DHS, a project that helps developing countries collect data on health and population trends) and record all the births that a surveyed woman has had and the age at death of any of her children who have died.

**Why Was This Study Done?:**

Because the DHS originally surveyed samples of 5,000–6,000 women, estimates of under-five mortality are traditionally calculated using data from five-year time periods. Over shorter periods with this sample size, the random errors in under-five mortality estimates become unacceptably large. Nowadays, the average DHS survey sample size is more than 10,000 women, so it should be possible to estimate under-five mortality over shorter time periods. Such estimates should be able to track the effects on under-five mortality of events such as droughts and conflicts better than estimates made over five years. In this study, the researchers determine appropriate time periods for child mortality estimates based on full birth histories, given different sample sizes. Specifically, they ask whether, with the bigger sample sizes that are now available, details about trends in under-five mortality rates are being missed by using the estimation procedures that were developed for smaller samples. They also ask whether calendar-year-based estimates can be calculated; mortality is usually estimated in “years before the survey,” a process that blurs the reference period for the estimate.

**What Did the Researchers Do and Find?:**

The researchers used a statistical method called “jackknife variance estimation” to determine coefficients of variance for child mortality estimates calculated over different time periods using complete birth histories from 207 DHS surveys. Regardless of the estimation period, half of the estimates had a coefficient of variance of less than 10%, a level of random variation that is generally considered acceptable. However, within each time period, some estimates had very high coefficients of variance. These estimates were derived from surveys where there was a small sample size, low fertility (the women surveyed had relatively few babies), or low child mortality. Other analyses show that although the five-year period estimates had lower standard errors than the one-year period estimates, the latter were affected less by bias than the five-year period estimates. Finally, estimates fixed to calendar years rather than to years before the survey were more directly comparable across surveys and brought out variations in child mortality caused by specific events such as conflicts more clearly.

**What Do These Findings Mean?:**

These findings show that although under-five mortality rate estimates based on five-year periods of data have been the norm, the sample sizes currently employed in DHS surveys make it feasible to estimate mortality for shorter periods. The findings also show that using shorter periods of data in estimations of the under-five mortality rate, and using calendar-year-based estimation, reduces bias (makes the estimations more accurate) and allows the effects of events such as droughts, epidemics, or conflict on under-five mortality rates to be tracked in a way that is impossible when using five-year periods of data. Given these findings, the researchers recommend that time periods shorter than five years should be adopted for the estimation of under-five mortality and that estimations should be pegged to calendar years rather than to years before the survey. Both recommendations have already been adopted by the United Nations Inter-agency Group for Child Mortality Estimation (IGME) and were used in their 2011 analysis of under-five mortality.

**Additional Information:**

Please access these websites via the online version of this summary at http://dx.doi.org/10.1371/journal.pmed.1001289.

- This paper is part of a collection of papers on Child Mortality Estimation Methods published in *PLOS Medicine*
- The United Nations Childrens Fund (UNICEF) works for children's rights, survival, development, and protection around the world; it provides information on Millennium Development Goal 4, and its Childinfo website provides detailed statistics about child survival and health, including a description of the United Nations Inter-agency Group for Child Mortality Estimation; the 2011 IGME report on Levels and Trends in Child Mortality is available
- The World Health Organization also has information about Millennium Development Goal 4 and provides estimates of child mortality rates (some information in several languages)
- Further information about the Millennium Development Goals is available
- Information is also available about Demographic and Health Surveys of infant and child mortality

## Introduction

Tracking Millennium Development Goal 4, which sets a target of reducing child mortality by two-thirds between 1990 and 2015, requires that the under-five mortality rate (also denoted in the literature as U5MR or _5_ *q*_0_), be estimated precisely and accurately. Moreover, child mortality is an important indicator of the performance of the public health system of a country. Demographic and Health Surveys (DHS) surveys and similar surveys that collect full birth histories are one of the chief sources of estimates of under-five mortality, deriving such estimates from women's own reports of their complete birth histories.

The DHS surveys were originally designed for samples of 5,000–6,000 eligible women [1]. Measures of child mortality have traditionally been reported for 5-y time periods, since time periods shorter than 5 y often result in sampling errors that are too large because estimates are based on the deaths that occur during the particular time spans. Many recent surveys have had sample sizes much larger than required by the original design, and these larger sample sizes may make it possible to use time periods shorter than 5 y for child mortality estimates.

Estimates for short time periods have the benefit that trends and deviations from trends may be ascertained with some detail. The mortality effects of particular events, such as a drought, hurricane, or the outbreak of war, may be tracked if the time period is sufficiently short. Moreover, recent major changes in the pace of change of child mortality may be discovered earlier than if long time periods are used for estimation.

The tracking of the Millennium Development Goals is partly global, but the responsibility for meeting the goals lies with each country. Moreover, regardless of international goal setting, countries need to monitor the development of their child mortality level as an important indicator of the performance of their public health system. Global tracking of child mortality can be considered to be served by the current 5-y period of estimation, since short-term and local effects might obscure the overall trend. However, the overall trends for the different countries are constructed by smoothing the data from several surveys [2], so the trend estimates can be relatively smooth even if the estimates from particular surveys reflect specific events or random fluctuations observed in a country. Thus, using short estimation periods in the estimation from individual surveys can serve both the overall trend analysis and the short-term concerns that are of interest for individual countries.

The term “time period” in the context of child mortality estimation actually has two meanings. The first is the duration of the period for which an estimate is derived, 1 y, 2 y, and so on. The second is somewhat subtle. The usual practice in DHS surveys is to estimate mortality based on the concept of “years before the survey.” That is, the estimation period is measured with reference to the time of the interview of each individual woman. As a consequence, the survey estimates for a period will actually cover a longer period than the apparent number of years in the period since the fieldwork may last from 2 mo to nearly a year. An alternative is to measure time in calendar years independent of when each woman was interviewed. The benefit of the traditional DHS approach is that it allows estimates into the fieldwork period of the survey, while the benefit of the calendar year approach is that it is provides a much clearer time reference. Thus, this paper will explore the consequences of using calendar-year-based estimates rather than estimates based on the time of the interview of each woman.

We proceed by considering criteria for when the sampling variability of an estimate is sufficiently small for tracking the levels and trends in child mortality, as well as the theory behind how to select appropriate time periods when sampling phenomena that fluctuated in time. We then give an overview of how DHS surveys are designed and how child mortality estimates and their sampling errors are computed. We then compute child mortality measures and their sampling errors for available DHS surveys in order to ascertain the conditions that allow the use of short time periods.

One way to determine appropriate time periods is to consider the standard errors of the estimates, and then reject any estimate if its standard error is too high. Since the standard error is scaled to what is measured, a common approach used in survey statistics and in other fields is to consider the coefficient of variation, i.e., the standard error divided by the estimate, expressed as a percentage or as the unmodified ratio [3],[4]. Strictly speaking, the coefficient of variation may be thought of as a population parameter, and as “relative standard error” or “coefficient of variation estimate” when calculated from samples [5]. Common usage is nevertheless to use the term “coefficient of variation” also for sample estimates.

It is commonly assumed that an estimate can be accepted for use or publication if its coefficient of variation is less than a given value, usually less than 5% or 10% of the estimate. Nevertheless, the rule of thumb of 5% or 10% is somewhat arbitrary, as is the case with many statistical rules of thumb, such as the 95% confidence interval [6]. Kish [7] agrees with Hansen et al. [8] that coefficients of variation larger than 0.2 (i.e., 20%) indicate a danger of unstable estimates, and notes that in his own practice “whenever the program found cv(x)>0.1 it printed out in red letters STOP, LOOK, AND DO SOMETHING” (“cv” is coefficient of variation) [7]. Hansen et al. [8] adopt 10% as a “working rule.” In a somewhat more bureaucratic tone, the Eurostat sampling guidelines require the national statistics offices of member states to ensure that the coefficient of variation is lower than 8% in reported estimates from national surveys [9].

Despite the fact that what exact level one should choose as a cutoff may be uncertain, the reason for not accepting high coefficients of variation is clear cut. Like the estimate of child mortality itself, the standard error as observed in a survey is a sample statistic. A large standard error (and correspondingly large coefficient of variation), means that one does not really know where within the confidence interval the true value lies, but only that the confidence interval is wide, because the statistics used to compute it are uncertain. When samples are small, both the estimates and their variance estimates become unstable. This was to a large extent Kish's concern in the quotation above. But when the coefficient of variation is smaller than the level implying instability, there is no obvious statistical reason for choosing between the conventional 5% or 10% or the Eurostat's 8%.

In addition to the purely statistical issue, there is a substantive issue of importance in choosing an acceptable level for the coefficient of variation. For a child mortality rate of 100 deaths per 1,000, a 10% coefficient of variation would give a 95% confidence interval of roughly 80–120, while a 20% coefficient of variation would give a 95% confidence interval of 60–140. The latter is way too large if one wants to estimate change from one period of estimation to the next, or indeed for estimating the level itself. But, ultimately, the question is one that revolves around what is needed for specific purposes.

The appropriate time period for analysis is not simply the smallest that the sample size can support. If one assumes the absence of bias in a given child mortality measure estimate, in principle the appropriate time period is determined by the level of child mortality, the level of fertility, the sample size (and the sample design), and the variation in child mortality over time. The Shannon-Nyquist sampling theorem [10] describes the relationship between the frequency of a signal and how often the signal needs to be measured in order to be accurately represented. The more frequent the variation, the shorter the period needed in order to accurately portray it. In general, if there is no fluctuation with a frequency higher than *B*, then one should sample at intervals that are shorter than 1/(2*B*). For example, to accurately describe mortality fluctuations that occur every second year, i.e., with a frequency equal to 0.5, one would need to sample with an interval no longer than a year (1/(2×0.5) = 1). The frequent sampling required by the sampling theorem is clearly unrealistic given the sampling error in the case of child mortality estimates.

**Figure 1:**
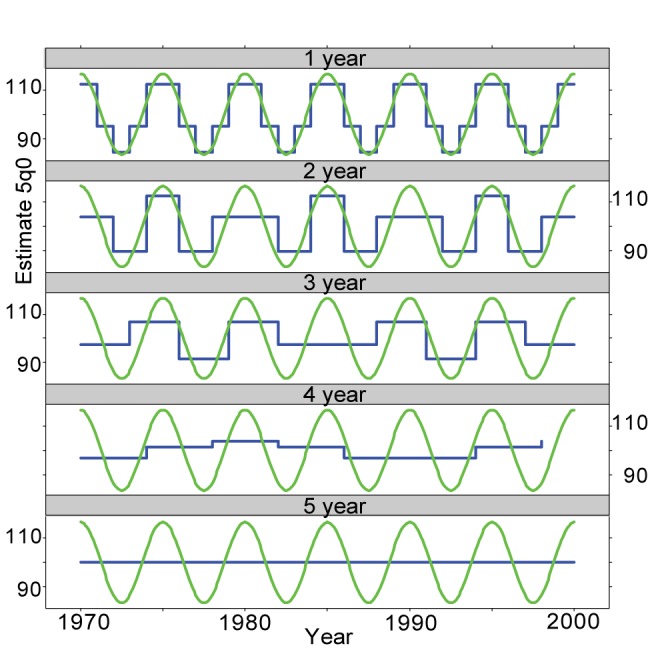
Effects of choice of period on estimates. The figure shows a cosine curve (green lines) with a 5-y periodicity and average estimates based on 1- to 5-y periods (blue lines).

The Shannon-Nyquist sampling theorem assumes measurements at instants. In the case of child mortality measurements, that is not possible, as child mortality estimates are, again for sample size reasons, not measured at instants, but for periods that are long compared to possible fluctuations in mortality level. If child mortality varies regularly with 5 y between peaks, then a series of 1-y periods of estimation is able to reconstruct the curve form relatively well, as shown by the step curve in Figure 1.

Even with a 1-y estimation period there is some distortion in terms of peaks and troughs, if one considers that child mortality is a continuous smooth function of time. With a 2-y period more major distortions appear, because the periodicity of the measurements does not match the periodicity of the variation. This is even more pronounced with 3- and 4-y periods, and with 5-y periods the original variation is not visible at all. The scenario shown in Figure 1, with a regular fluctuation of mortality, may be that which will be most distorted, since the regularity forces long periods to average out fluctuation. With a 5-y period, a peak during a single year or two followed by a return to a baseline will be detectable, although the importance of the peak will be understated.

**Figure 2:**
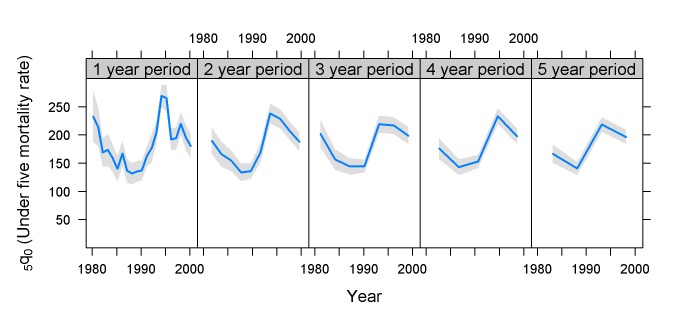
_5_ *q*_0_ and confidence intervals for the Rwanda 2000 DHS survey. Estimates based on years before the survey.

The Rwanda 2000 DHS survey provides an empirical example of the issues at hand. The birth history recorded in the survey covers the 1994 genocide. Although the massacres took place mainly from April to July, effects on child mortality may have lasted longer. As can be seen in Figure 2, while the 1994 events are somewhat detectable on the 5-y period plot, the effect is clearly visible on the 1-y period plot, showing that the 5-y estimates seriously understate the mortality increase, even when one disregards selection effect issues such as that children whose mothers were killed, and who therefore were not interviewed in 2000, must have had a much higher risk of death than other children. The 2-y trend line also shows a substantial flattening of the trend compared to the 1-y trend, illustrating empirically the theoretical plot in Figure 1.

The distortion of the estimates is a problem that pertains not only to the shape of the trend lines of the mortality measures, but also—as the literature within economics indicates—to the issue that the coefficients of models based on the long time periods may be biased [11], in a way that is similar to misspecification error or ecological fallacy. This is a problem for models that use aggregated child mortality measures, such as attempts to create models of the relationship between gross domestic product per capita and child mortality for countries (e.g., [12],[13]) or models that aim to describe the relationship between conflict and child mortality levels (e.g., [14],[15]), but not for models focusing on finding covariates for individual child deaths.

A few observations follow from this exposition. First, sample sizes and mortality levels constrain how short a period can be used for estimation of child mortality measures. Second, having a large sample and using it to estimate child mortality measures with long observation periods is not optimal, because if mortality fluctuates, then significant bias may be a result. Thus, it may be better to trade lower precision (higher variance) in the estimate for higher accuracy (reduced bias) than to assume that a 5-y estimation period is always preferable. The problem remains, however, how that trade-off should be calibrated, and that will be addressed in the rest of this article. The purpose of this article is to determine appropriate time periods for child mortality estimates from full birth histories, given various sample sizes. Thus, it tries to answer the question of whether, given the increased sample sizes in recent years, we are wasting information by sticking to the estimation procedures that were developed for smaller samples.

## Methods

### Data Sources

The data used in this analysis were datasets that were publicly available from DHS, i.e., they could be downloaded from the Measure DHS website (http://www.measuredhs.com/data/available-datasets.cfm) without special permission from the country in question. Surveys that were carried out after 1 January 1985 and had data released before 31 October 2010 were included (surveys before 1990 do not appear to include sampling stratum information; a complete list of the surveys used is in Table S1). The data were not edited or altered in any way, except in the case where a dataset had one or more sampling strata consisting of only one cluster. In such cases each stratum in question was merged with an adjacent stratum or with a nearby stratum that also had only one cluster, i.e., employing the so-called collapsed stratum technique [16]. This will tend to overstate variance slightly. In some cases we did not feel confident that we had understood the stratification of the survey properly (e.g., Egypt 2005), and such surveys were dropped from the analysis. In total, 207 surveys were retained for analysis.

Rather than using exact dates in the estimation, DHS uses so-called century month coding, in which dates are converted into the number of months since January 1900. Some surveys, such as the Nepali and Ethiopian ones, use national calendars. These were recalculated according to the standard DHS coding and the Gregorian calendar. DHS data files do not contain the day part of dates, and therefore the conversion can be only approximate.

### DHS Survey Design

The DHS surveys generally employ a two-stage stratified sampling design [1]. The sample is usually explicitly stratified by major administrative regions of the country in question, and also by urban and rural location. The allocation of the sample varies, and may be disproportionate in order to ensure adequate reporting domains, although proportionate allocation is preferred. The samples are very often implicitly stratified by geographic location within the explicit strata. Primary sampling units (PSUs) are most often census enumeration areas, consisting of 50 to 300 households. They are selected with probability proportionate to their size, as measured by their number of households, usually derived from the latest census of the country in question. If the PSUs are large then they are usually segmented, and only one segment is selected with probability proportionate to its number of households. PSUs (or segments) are always mapped, and a current list of households is created before the selection of households takes place by linear systematic sampling. Depending on the survey, and sometimes on geographic location within a specific survey, about ten to 20 households are selected in each PSU. As noted above, DHS surveys were designed for a sample size of 5,000–6,000 eligible women or approximately the same number of households, but many surveys have had larger samples, with those of India and Pakistan being the largest, with more than 100,000 households. Since 2005, the median sample size has been over 10,000 households, corresponding to an increase of roughly 3,000 households since 1990–1994 (Table 1).

**Table 1:** Sample Sizes for DHS surveys, 1985–2010.

Year	All Surveys						Surveys with Samples<20,000 Households					
	Sampled Households			Eligible Women			Sampled Households			Eligible Women		
	Mean	Median	Count	Mean	Median	Count	Mean	Median	Count	Mean	Median	Count
**1985–1989**	6,824	6,268	28	6,057	5,532	28	6,824	6,268	27	6,057	5,532	27
**1990–1994**	11,640	7,354	34	11,024	6,929	34	7,662	6,709	31	7,391	6,864	31
**1995–1999**	11,003	8,016	54	10,947	8,298	54	8,079	7,922	46	8,287	8,048	46
**2000–2004**	12,640	10,259	49	11,964	10,505	49	10,549	9,884	38	10,388	9,157	38
**2005–2010**	17,903	10,310	61	15,662	10,401	61	10,010	9,720	44	9,969	9,326	44
**Total**	12,728	8,975	226	11,781	8,337	226	8,789	8,106	186	8,641	7,958	186

Based on output from the Measure DHS STATcompiler, and relevant survey reports when data were unavailable from the compiler.

Sampling weights are calculated so that they account for the details of the design. Although the DHS design is in principle self-weighting within the explicit strata, weight variations do occur because the estimate of the PSU sizes from the sampling frame may differ from the actual number found in the PSUs during the mapping and listing. Moreover, weight variation naturally occurs between strata that have different overall sampling rates.

### Mortality Estimation from Full Birth Histories

Full birth histories are taken from all women aged 15–49 y in most surveys, although some, principally in the Middle East and southern Asia, define only married women in the 15- to 49-y-old group as eligible. Both usual residents of the households and visitors are used in the estimation.

The DHS estimates of child mortality are calculated using a synthetic cohort approach [17]. The age span from 0 to 5 y is split into eight groups, 0 to 30 d and 1 to 2, 3 to 5, 6 to 11, 12 to 23, 24 to 35, 36 to 47, and 48 to 59 mo. Survival probabilities from the beginning to the end of each age group are calculated, and then the appropriate measures are calculated as the product of the composite age rates.

### DHS Variance Estimates

Estimates of variance for child mortality estimates from full birth histories collected through a typical household survey are somewhat involved. That is both because the various mortality ratios are computed in a complex fashion as described above, so that working out their variance estimator would be practically impossible, and because the sampling design is complex, i.e., includes unequal weights, clustering, and stratification, resulting in estimators that are not linear.

The variance estimates in the DHS reports have been calculated with a jackknife procedure. The basic premise of this method is that the realized sample can be seen as a good approximation of the population distribution, and that repeated samples from the sample can be made. The distribution of the estimates made from these subsamples will then reflect the sampling variability. While several methods based on this premise exist, the unique characteristic of the jackknife approach is that subsamples are formed by deleting one sampling unit from the sample, calculating the estimate, replacing the deleted unit, and then repeating the procedure with the next sampling unit. Thus, a number of replicate estimates equal to the number of sampling units is formed, and the variance is basically the variance of the distribution of replicates.

In jackknife cluster samples, the ultimate clusters, rather than the last stage sampling units, are used. Thus, for example, with a sample of 368 clusters and 24,000 births, 368 replicates are computed, not 24,000.

According to the DHS sampling manual [1] and the various country reports, the formula used for the variance estimate is
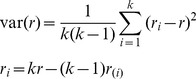
(1)where *k* is the number of ultimate clusters, *r* is the estimate from the full sample, and *r*_(*i*)_ is the estimate from the reduced sample of *k*−1 clusters, when cluster *i* is excluded.

A characteristic of the variance estimator as employed by DHS is that it disregards stratification. Therefore, it likely overestimates variance, at least in samples where strata allocations are more or less proportional to the stratum sizes. A test on the 2002 DHS survey for Eritrea confirms that it was indeed the unstratified estimator that was used.

An alternative variance estimator is the JKn variance estimator used by Westat's WesVar software [18] and by SUDAAN [19], and described by Wolter [16]. The stratified JKn procedure for estimating variance is very similar to that of the unstratified version, i.e., it deletes and replaces each ultimate cluster in turn. However, when a cluster is omitted, the weights of the other clusters in the stratum are adjusted by a factor of *n_h_*/(*n_h_*−1), where *n_h_* is the number of clusters in the stratum. Then, the actual variance estimate is calculated as given below, where *h_k_* is (*n_h_*−1)/*n_h_*:
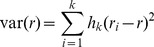
(2)Note that in this case *r_i_* is the actual estimate from the *i*-th replicate, i.e., without the transformation applied in the formula used by DHS. The DHS formula and Equation 2 are in fact equivalent: if the adjustment to the weights and the *h_k_* factor are not applied, the two formulas yield the same result. Thus, the difference is the adjustment for stratification. The formula that DHS uses is the original version as described by Quenouille, the inventor of the method [16]. Neither the DHS version of the variance estimate nor the stratified version includes a finite population correction. In general, sampling rates in DHS surveys are too small for this to matter.

The differences in standard errors between the estimates that DHS presents and those taking stratification into account are on the order of 10% or less, since the variances of the child mortality measures usually do not differ all that much between strata.

The mortality estimates and standard errors were produced by a program written in Delphi (Embarcadero RAD Studio XE). For each data source, estimates for 1-, 2-, 3-, 4-, and 5-y periods were calculated as far back in time as was possible for each survey, given the length of the birth histories. The estimates of childhood mortality for 5-y periods before the survey match exactly those produced by the Measure DHS online statistics compiler (STATcompiler) or the DHS final reports for datasets that are not represented in the compiler output. Delphi was chosen because the system produces compiled code. It is therefore much faster than the interpreted code used by R, Stata, or SPSS. The program reads comma-separated or Excel files exported from the original DHS data files. It is available from the authors on request. Computed estimates from the DHS surveys used in our analysis are found in Tables S2 and S3.

## Results

**Figure 3:**
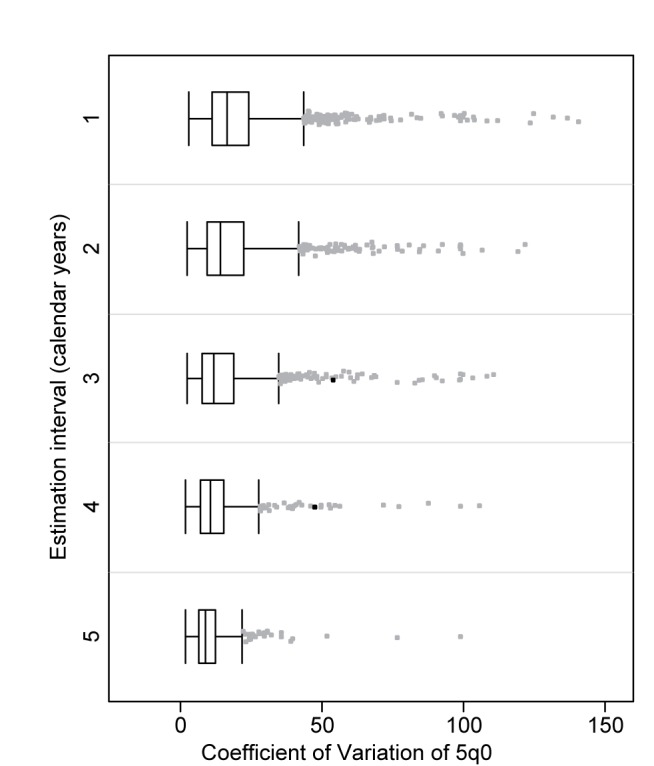
Distribution of coefficient of variation of _5_ *q*_0_ by length of period of estimation. Only estimates for periods 20 y or less before the survey were included. Two extreme outliers have been excluded from the graph.

Regardless of the length of the estimation period, 75% of the estimates of _5_ *q*_0_ have a coefficient of variation below 14%, and half below 10%. For estimation for periods of 3 y and more, the estimate of the coefficient of variation is less than 10% in 75% of the cases (Figure 3; Table 2). Nevertheless, the distribution within each period is highly skewed, with a few very high coefficients. In general, the outliers are surveys that have small sample sizes, represent estimation periods long before the survey date, have low fertility, or have low mortality, but there are some exceptions to this pattern.

**Table 2:** Distribution of coefficient of variation for _5_ *q*_0_ by length of period.

Period of Estimate	Mean	25th Percentile	Median	75th Percentile
1 y	0.13	0.07	0.10	0.14
2 y	0.10	0.06	0.08	0.11
3 y	0.09	0.05	0.07	0.10
4 y	0.08	0.05	0.06	0.09
5 y	0.07	0.04	0.06	0.08
Total	0.10	0.06	0.08	0.12

Calendar-year estimates. Only estimates 20 or fewer years before the survey included.

**Figure 4:**
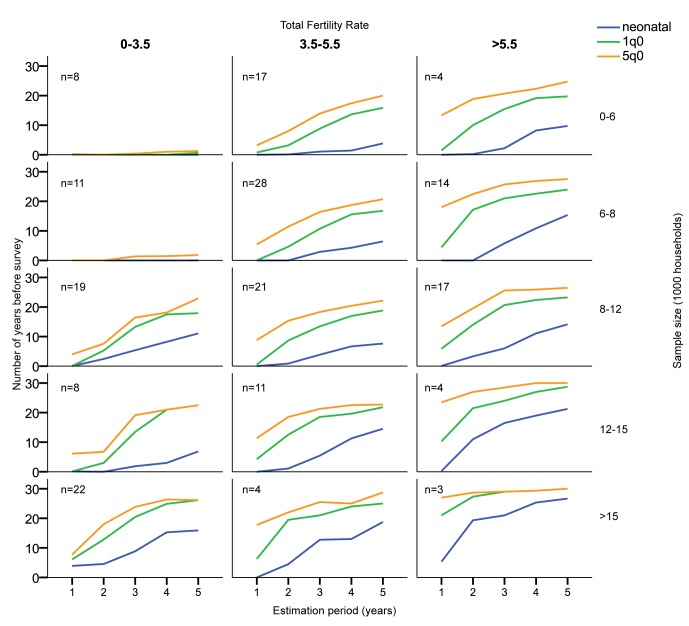
Mean number of contiguous years before survey allowing estimates with coefficient of variation less than 10%, by sample size, estimation period, and total fertility rate (births per woman). Estimates based on years before the survey. The number of surveys used for the estimates in each panel is indicated in the upper left corner of each.

**Figure 5:**
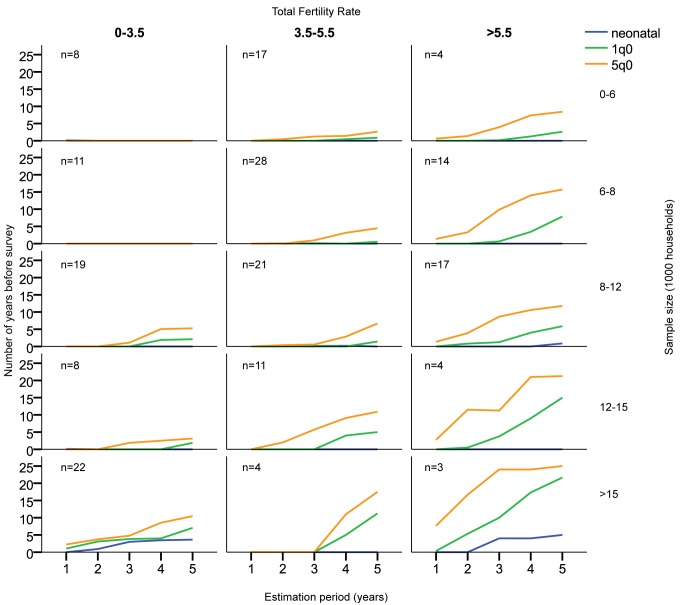
Mean number of contiguous years before survey allowing estimates with coefficient of variation less than 5%, by sample size, estimation period, and total fertility rate (births per woman). Estimates based on years before the survey. Estimates based on calendar years. The number of surveys used for the estimates in each panel is indicated in the upper left corner of each.

The results are highly determined by sample size, mortality level, and fertility (Figures 4 and 5; Table S1). To some extent, fertility can be used as a proxy for mortality level, since countries with high fertility usually also have high child mortality. In general, with the total fertility rate at 5.5 births per woman or higher, all realistic sample sizes, including small ones, make the estimation of _5_ *q*_0_ for single-year periods at least 10 y back from the survey date feasible if a coefficient of variation of less than 10% is the criterion. At medium fertility levels, 3.5–5.5 births per woman, one would need a sample of at least 8,000 to be able to estimate 5 y back with 1-y periods, while 12,000 would be required for estimating 10 y back. At fertility levels lower than 3.5 births per woman, estimation of _5_ *q*_0_ with samples of less than 8,000 households appears to be ill-advised, regardless of the period for estimation. At low fertility levels and with small samples, the only estimate that can be made with confidence in most cases is _5_ *q*_0_ for 0–4 y before the survey, and sometimes not even that (e.g., Kazakhstan).

One should note, however, that there are exceptions to the general rules of sample size and fertility. Sierra Leone 2008 has, for example, no estimate with a coefficient of variation lower than 10%. It is unclear why this is so, since Sierra Leone 2008 has a standard DHS sample design, with a sample size of 7,572 households and a total fertility rate of 5.1 births per woman.

Similar considerations pertain to neonatal mortality (formally, the number of deaths during the first 28 d after birth per 1,000 births, but estimated in DHS surveys and here as the number of deaths during the first month) and infant mortality (the number of deaths during the first year after birth per 1,000 births) estimates. Since the number of deaths observed in a period is smaller for these estimates, the number of years with low coefficients of variation is also fewer than for the _5_ *q*_0_ estimates. Nevertheless, if a coefficient of variation of less than 10% is the standard, then it is possible to produce mortality estimates 5 to 10 y back, even for neonatal mortality in high-fertility populations for most samples, but estimation further back than the most recent 5 y is likely to be unstable.

Not surprisingly in the case of neonatal mortality, the number of continuous years before the survey that can provide estimates with acceptable coefficients of variation is quite small regardless of the period used. For 1-y estimation periods, one needs samples of more than 15,000 households to be able to estimate back much in time.

If a coefficient of variation of less than 5% is the standard, then samples of more than 15,000 households are needed in order to make estimates for 1-y periods for _5_ *q*_0_, and neonatal and infant mortality can seldom be estimated at all (Figure 5; Table S1).

**Figure 6:**
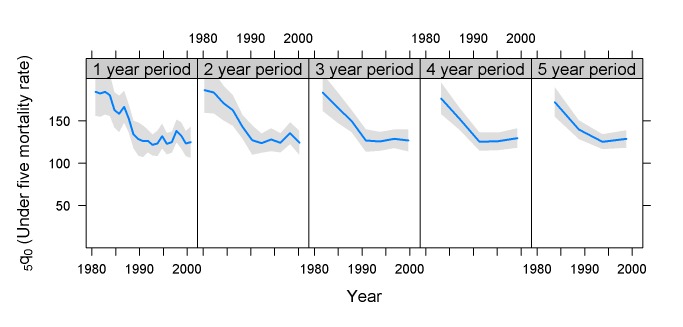
Estimates of _5_ *q*_0_ and confidence intervals for Mali 2001 DHS survey. Sample size 12,439 households; total fertility rate 6.8 (births per woman). Estimates based on years before the survey.

The results described above can also be seen graphically for each individual country survey. The general trend, regardless of sample size, is that the confidence intervals around the curves are quite narrow close to the survey date, and they then become wider the further away from the survey date the estimate is located. Mali 2001 (Figure 6) is an example of a survey of a high-mortality and high-fertility situation (_5_ *q*_0_ of 200/1,000 or more; total fertility 5 y before the survey was 6.8 births per woman), with a relatively large sample (13,429 completed household interviews). Such a survey allows estimation for 1-y periods more than 20 y back in time using the 10% criterion. It is also a case where the period of estimation makes little difference, because the development of mortality has been relatively smooth.

**Figure 7:**
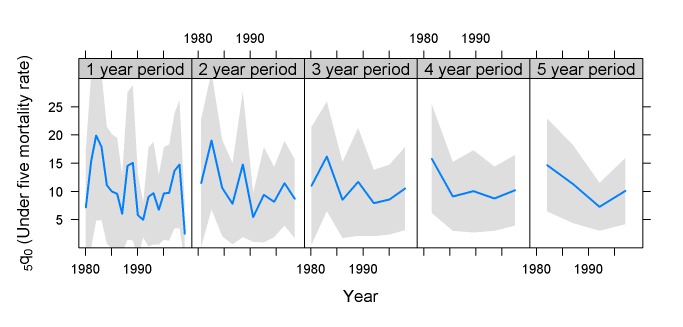
Estimates of _5_ *q*_0_ and confidence intervals for Kazakhstan 1999 DHS survey. Sample size 5,841households; total fertility rate 2.0 (births per woman). Estimates based on years before the survey.

In contrast to Mali 2001, the Kazakhstan 1999 survey had a small sample, and the country had relatively low mortality and quite low fertility (Figure 7). The survey estimates have generally wide confidence intervals. The 1- and 2-y estimates suggest a peak in child mortality around 1997 and then a decline, while the 5-y line suggests a slight recent increase.

**Figure 8:**
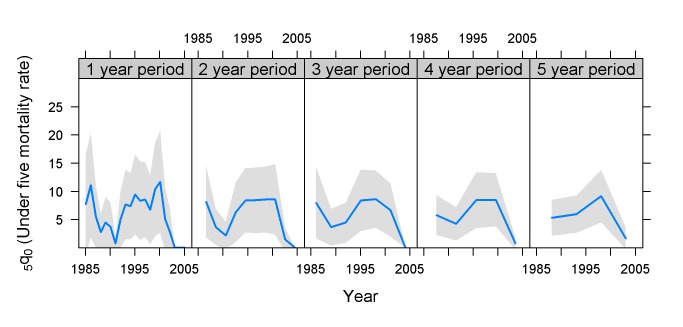
Estimates of _5_ *q*_0_ and confidence intervals for Moldova 2005 DHS survey. Sample size 11,095 households; total fertility rate 1.7 (births per woman). Estimates based on calendar years.

Moldova 2005 (Figure 8) has twice the sample size of Kazakhstan 1999, and this is reflected in the more narrow confidence bands. Still, it is difficult to achieve really small coefficients of variation for surveys in countries with relatively low mortality and very low fertility.

**Figure 9:**
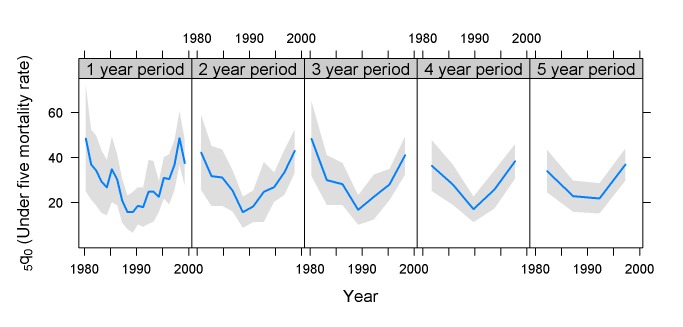
Estimates of _5_ *q*_0_ and confidence intervals for Zimbabwe 1999 DHS survey. Sample size 6,372 households; total fertility rate 4.0 (births per woman). Estimates based on calendar years.

The Zimbabwe 1999 data show a characteristic trumpet shape of the confidence bands, which is seen in many surveys: the further the estimate is from the survey date, the wider the estimate of the confidence band (Figure 9). The reason for this is that for estimates back in time, only a portion of the women interviewed provide data. Thus, children reported for 20 y ago were born to women who at the time were under 30 y. This reduces the sample size, and also introduces selection biases, since such estimates reflect the mortality of children born only to young mothers, and because it excludes children of women who have died.

### Issues of Bias

As noted in the Introduction, focusing on the coefficient of variation overlooks the combined effects of bias due to long periods and sampling error. In principle, one can consider the mean square error, rather than the sampling error in isolation. If the bias and sampling error are uncorrelated, then the mean square error (MSE) is given by:
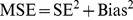
(3)The square root of the mean square error, usually termed root mean square error, is then comparable to a standard error, but includes the effect of the bias. Thus, the root mean square error is a measure of the total uncertainty associated with the estimate.

The problem is to estimate the bias. A simple approach is to assume that the difference between two estimates, e.g., for a 5-y period and a 1-y period, constitutes the bias of the longer period. That is certainly inaccurate, not the least because both estimates are subject to sampling error, and the 1-y estimate also may have some long-period bias. Moreover, the 1-y estimate is also biased because of selection effects, as noted before. With these caveats, one may reconsider the calculation of the root mean square error for the _5_ *q*_0_ mortality trend during the genocide period in Rwanda, as recorded by the 2000 DHS survey.

The root mean square error for the 5-y estimate (using the difference between the 1-y estimate for February 1994 and the 5-y period centered on February 1993 as a proxy for bias) is 51.2, while that for the February 1994 1-y period estimate remains 10.4, as the bias is assumed to be 0 (Table 3).

**Table 3:** Single-year and 5-y estimates of _5_ *q*_0_ and standard errors for the Rwanda 2000 DHS survey.

Midpoint of Reference Period	Single-Year Estimate			5-y Estimate		
	_5_ *q*_0_	SE	CV	_5_ *q*_0_	SE	CV
1986 February	167.3	11.8	7.0%			
1987 February	136.2	10.7	7.9%			
1988 February	131.5	9.4	7.2%	140.5	6.0	4.3%
1989 February	135.4	9.6	7.1%			
1990 February	136.9	8.9	6.5%			
1991 February	162.2	9.2	5.7%			
1992 February	177.1	12.9	7.3%			
1993 February	204.8	10.1	4.9%	218.6	6.7	3.0%
1994 February	269.3	10.4	3.9%			
1995 February	265.2	11.9	4.5%			
1996 February	191.9	9.9	5.1%			
1997 February	194.1	10.2	5.3%			
1998 February	219.8	10.1	4.6%	196.2	6.5	3.2%
1999 February	195.6	11.4	5.8%			
2000 February	179.9	11.0	6.1%			

Estimates based on years before survey.

CV, coefficient of variation; SE, standard error.

**Figure 10:**
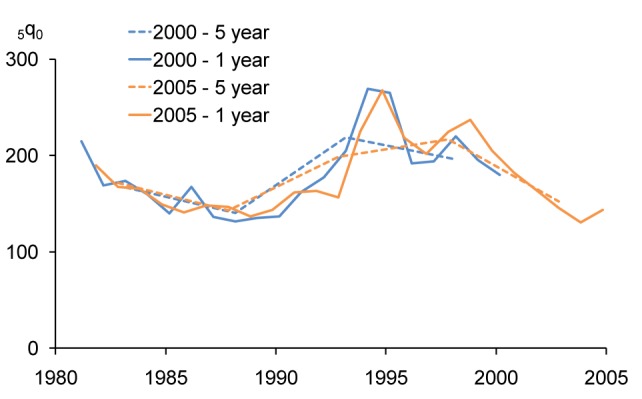
Trends in _5_ *q*_0_ in Rwanda 2000 and 2005 DHS surveys using estimation periods based on years before the survey.

The large root mean square error of the 5-y estimate appears credible when considered together with a graphical view of the development of _5_ *q*_0_ in Rwanda (Figure 10), with the data from the 2000 and 2005 DHS surveys (the 2007–2008 DHS survey shows a very different trend, and is disregarded here, while the 1992 DHS survey is consistent with the 2000 and 2005 surveys but irrelevant for the genocide period). A first observation that can be made from Figure 10 is that the 1-y period estimates from both the 2000 and 2005 surveys show clear peaks in 1994, although the 2005 survey shows one peak between the two peaks of the 2000 survey—most likely because of the difference in exactly when the time period of the estimate is centered. A second observation from the graph is that to some extent 1-y periods may remove some of the inconsistencies associated with trends based on the 5-y period estimates. The 5-y periods from both surveys show a leveling off of increasing mortality around the time of the genocide. The 2000 DHS survey suggests that this happened before the genocide, while the 2005 survey suggests that it happened after.

The preceding analysis implicitly makes three points. First, even though the 5-y period estimates in general have lower standard errors than the 1-y period estimates, the 1-y period estimates may be preferable, because of the potential bias that may occur in using the 5-y estimates. Second, one possible decision criterion is that when the mean square error (as calculated above) is larger for the 5-y estimate than for the 1-y estimate, the 1-y estimate is preferable. Third, if the coefficient of variation of the 1-y estimate is sufficiently low (i.e., at least lower than 10%), then the 1-y estimate should be preferred. In cases where the differences between the 5-y period estimates and the 1-y estimates are slight, the choice is largely irrelevant.

A further consideration is the time location of the estimates. As noted, the DHS estimates cover a longer calendar period than the estimation period, because the time reference is with respect to each individual interview date. This is a relatively minor consideration for 5-y periods, especially if the fieldwork period is short, but becomes an important consideration for the short estimation periods, such as 1- and 2-y periods, especially in the context of rapidly changing mortality. The consideration is also becoming increasingly important as samples increase in size, since large surveys require longer fieldwork periods.

**Figure 11:**
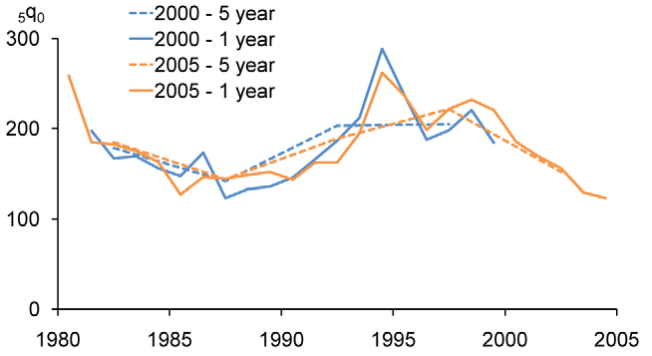
Trends in _5_ *q*_0_ in Rwanda 2000 and 2005 DHS surveys using calendar-year estimation periods.

The effect of the time location can easily be seen by recomputing the estimates for calendar years rather than years before the survey. The midyear estimates of _5_ *q*_0_ computed from the Rwanda 2000 and 2005 surveys were, respectively, 208 and 192 for 1993, 281 and 260 for 1994, and 248 and 235 for 1995, i.e., a match that puts the yearly estimates squarely within each other's confidence intervals, and with peaks in the same years (Figure 11).

Since the Rwanda massacres took place within a calendar year, it is likely that estimates based on calendar year are more accurate than estimation based on years before the survey. But what works best for sudden changes in mortality is dependent on the actual time location of the sudden change. Nevertheless, fixing the estimates to calendar years rather than to years before the survey, which is anchored in the individual interview dates of the women providing the birth histories, has the benefit of making the estimates more directly comparable across surveys. The apparent inconsistencies between the 1-y estimates from the two Rwanda surveys, as well as between the 5-y period estimates, disappear with calendar-year-based estimation.

## Discussion

Our analysis shows that whereas childhood mortality estimates based on 5-y periods of data have previously been the norm, the sample sizes presently employed in DHS surveys make it feasible to base the estimates on shorter periods. It also shows that using calendar years rather than years before the survey as a basis for estimation is useful because it brings out variation in child mortality in a clearer and more realistic manner.

Apart from the benefit in relation to tracking the Millennium Development Goals and the general benefit of avoidance of bias, there are three main benefits of this approach in relation to monitoring of public health. The first is that changes in mortality rates may be detected earlier. The second is that the relationship between child mortality rates and other events may be elucidated. One example is that of Rwanda, where the use of 5-y rates based on years before the survey seriously distorts the effect of the genocide on child mortality. Another example is the relationship between child mortality and outbreaks of some epidemic diseases, such as measles, which may be detected with the use of 1-y rates, but which will be undetectable with the use of 5-y rates. Finally, using short time periods for estimates, survey data may be used to establish consistency with other sources of child mortality estimates. Vital registration is often incomplete in developing countries, but one often assumes that the trends in vital registration data can be used for tracking fluctuation in child mortality [20]. This assumption is difficult to check with 5-y estimates from survey data, since vital registration typically provides data for single years, but the check can be carried out with single-year estimates.

For surveys with sample sizes larger than 8,000 households, it is possible in most cases to estimate child mortality for single years about 10 y back before the survey, although for low-fertility countries this cannot be done. For really large sample sizes, i.e., 20,000 or more, the factor limiting estimation for single-year periods is more the inherent selection bias when estimating back in time, than the sample size itself.

How short should the estimation period be? The results for countries such as Rwanda, with a very abrupt mortality change, suggest that 2-y periods do not bring large improvement to the estimation compared to 5-y periods, because somewhat unpredictable misestimation may still occur.

A practical rule for when short time periods should be chosen is when the mean square error (as calculated above) for the 5-y estimates becomes large compared to that of the short-period estimates. Large mean square errors are likely to occur when there are rapid surges or drops in mortality. In practice, one can use the same decision rule for the relative mean square error as for the coefficient of variation. A simpler decision rule is to use estimates with a coefficient of variation lower than 10%.

Some of the variation in child mortality estimates between different surveys in a population with changing rates of change in mortality stems from the way time periods are calculated. Both for 5-y periods and shorter ones, there is merit in changing estimation to fixed calendar years, rather than years before the survey. This is particularly the case for short time periods, but may likely also resolve some of the differences between survey estimates from different surveys in the same country, even for 5-y periods.

The two main recommendations given here—adopting shorter time periods than 5 y for estimation and using calendar years—have been adopted by the United Nations Inter-agency Group for Child Mortality Estimation in the 2011 estimates of child mortality [21].

## Supporting Information

Table S1 Number of contiguous years from survey date allowing estimates of mortality with coefficient of variation less than 10%.
(XLS)

Table S2 Estimates of mortality and standard errors, part 1: Albania to Jordan.
(XLS)

Table S3 Estimates of mortality and standard errors, part 2: Jordan to Zimbabwe.
(XLS)

## Abbreviations

_5_q_0_: under-five mortality rate
DHS: Demographic and Health Surveys
PSU: primary sampling unit
